# Introgression in the genus *Campylobacter*: generation and spread of mosaic alleles

**DOI:** 10.1099/mic.0.045153-0

**Published:** 2011-04

**Authors:** Samuel K. Sheppard, Noel D. McCarthy, Keith A. Jolley, Martin C. J. Maiden

**Affiliations:** Department of Zoology, University of Oxford, South Parks Road, Oxford OX1 3PS, UK

## Abstract

Horizontal genetic exchange strongly influences the evolution of many bacteria, substantially contributing to difficulties in defining their position in taxonomic groups. In particular, how clusters of related bacterial genotypes – currently classified as microbiological species – evolve and are maintained remains controversial. The nature and magnitude of gene exchange between two closely related (approx. 15 % nucleotide divergence) microbiologically defined species, *Campylobacter jejuni* and *Campylobacter coli*, was investigated by the examination of mosaic alleles, those with some ancestry from each population. A total of 1738 alleles from 2953 seven-locus housekeeping gene sequence types (STs) were probabilistically assigned to each species group with the model-based clustering algorithm structure. Alleles with less than 75 % assignment probability to one of the populations were confirmed as mosaics using the structure linkage model. For each of these, the putative source of the recombinant region was determined and the allele was mapped onto a clonalframe genealogy derived from concatenated ST sequences. This enabled the direction and frequency of introgression between the two populations to be established, with 8.3 % of *C. coli* clade 1 alleles having acquired *C. jejuni* sequence, compared to 0.5 % for the reciprocal process. Once generated, mosaic genes spread within *C. coli* clade 1 by a combination of clonal expansion and lateral gene transfer, with some evidence of erosion of the mosaics by reacquisition of *C. coli* sequence. These observations confirm previous analyses of the exchange of complete housekeeping alleles and extend this work by describing the processes of horizontal gene transfer and subsequent spread within recipient species.

## INTRODUCTION

The availability of multi-locus genetic data from large collections of bacterial isolates has demonstrated the central role of horizontal genetic exchange in the evolution of many bacterial populations (Didelot & Maiden, 2010; Fraser *et al.*, 2007). The extent of this process varies from essentially none, in clonal monomorphic bacteria such as *Mycobacterium tuberculosis* (Gutacker *et al.*, 2006) and *Salmonella* Typhi (Kidgell *et al.*, 2002), to extensive in *Neisseria meningitidis*, *Streptococcus pneumoniae*, *Staphylococcus aureus* (Feil & Spratt, 2001) and *Campylobacter jejuni* (Sheppard *et al.*, 2008; Wilson *et al.*, 2009), and extreme in *Helicobacter pylori*, which has a non-clonal population structure (Falush *et al.*, 2001). Horizontal acquisition of genes influences bacterial ecology and evolution (Ochman *et al.*, 2000), for example by increasing the rate at which favourable mutations accumulate within a population (Baltrus *et al.*, 2008; Cooper, 2007), or by conferring novel metabolic capabilities (Lawrence, 1999; Spratt *et al.*, 2001), and in recombinogenic species it can be more important than mutation in determining the genome content (Feil & Spratt, 2001; Ochman & Groisman, 1994).

Genetic exchange was first demonstrated in the bacteria in the 1940s (Lederberg & Tatum, 1946), but it was with the widespread application of DNA sequencing methods at the end of the 20th century that its extent and evolutionary significance were appreciated, challenging the concept that all bacteria were essentially asexual organisms (Maynard Smith *et al.*, 2000). Multi-locus sequence typing (MLST) (Maiden, 2006), and related techniques, demonstrated that, notwithstanding high frequencies of genetic exchange in many bacterial populations, seven-locus allelic profiles (sequence types, STs) contained sufficient information to associate genotype clusters with phenotypic properties commonly associated with microbiological species and subspecies groups (Hanage *et al.*, 2005; Maiden, 2006; Sheppard *et al.*, 2008).

In addition to the reshuffling of intact genes, intragenic recombination between genetically divergent loci can generate new ‘mosaic’ alleles. These are alleles where portions of the nucleotide sequence have different evolutionary histories combined in a single gene allele (Maynard Smith *et al.*, 1991; Milkman & Stoltzfus, 1988). Mosaic alleles, which are readily generated in bacteria because RecA-mediated DNA repair systems can accept the import of DNA with up to 30 % sequence divergence (Lorenz & Wackernagel, 1994), have been described in genera as diverse as *Streptococcus* (Hollingshead *et al.*, 2000; Kapur *et al.*, 1995), *Neisseria* (Maiden *et al.*, 1996; Maynard Smith *et al.*, 1991; Spratt, 1988; Spratt *et al.*, 1991) and *Escherichia* (Milkman & Stoltzfus, 1988; Stoltzfus *et al.*, 1988). Many studies have focused on mosaic genes that have obvious phenotypic effects, such as increased antibiotic resistance (Coffey *et al.*, 1995; Dowson *et al.*, 1990), virulence (Halter *et al.*, 1989) and antigenic variation (Snyder *et al.*, 2007), where positive selection can be invoked to explain the spread of rare novel variants in a population; however, mosaic alleles have also been described among housekeeping genes that are subject to stabilizing selection (Dingle *et al.*, 2005; Zhou & Spratt, 1992).

Because the donor and recipient regions of the mosaic housekeeping genes can potentially be identified, they can provide information on the patterns of gene flow in the absence of overt strong positive selection (Brückner *et al.*, 2004; Hakenbeck, 1998; Maynard Smith *et al.*, 1991; Milkman & Stoltzfus, 1988; Maynard Smith, 1992; Stoltzfus *et al.*, 1988). Using mosaic alleles to investigate gene flow has advantages over whole-allele replacements (Hanage *et al.*, 2005; Sheppard *et al.*, 2008), firstly because the potential for incorrectly defined mixed STs (Caro-Quintero *et al.*, 2009) is removed, and secondly because the recombination profile representing a single genetic event can be traced through the population to determine patterns of recombination. This allows the different types of hybrid proliferation to be differentiated, including founding events, clonal expansion and within-species horizontal transfer.

Recombination, and barriers to it, have been identified as an important force in determining bacterial population structure (Hanage *et al.*, 2006; Lawrence, 2002). However, many fundamental parameters have yet to be established and the relationship of bacterial population structure to ‘species’ remains a matter of debate (Cohan & Koeppel, 2008; Doolittle, 2008; Fraser *et al.*, 2009). Large-scale nucleotide-sequence-based population studies permit the determination of the relative rates and mechanisms of gene flow among populations that are necessary to test the various theoretical paradigms and to investigate the role of neutral and selective forces in these processes (Fraser *et al.*, 2009). In this study, we investigated bacterial ‘introgression’ – the movement of DNA between recognized species – in the species *Campylobacter jejuni* and *Campylobacter coli*, which together represent the major cause of bacterial gastroenteritis in many parts of the world (Sheppard *et al.*, 2009). Mosaic alleles among *C. jejuni* and *C. coli* genotypes were characterized and used to investigate the movement and spread of genetic material between these two closely related taxa (Friis *et al.*, 2010), confirming the observations made on the exchange of whole MLST loci (Sheppard *et al.*, 2008), and demonstrating the pattern of horizontal gene transfer and the fate of transferred genetic material.

## METHODS

### Genotype data.

*Campylobacter jejuni* and *Campylobacter coli* genotype information was obtained from the publicly accessible MLST database (http://pubmlst.org/campylobacter) (Jolley *et al.*, 2004). The *C. jejuni* and *C. coli* data archive contains ST information for seven housekeeping loci: *aspA*, *glnA*, *gltA*, *glyA*, *pgm* (*glmM*), *tkt* and *uncA* (*atpA*), positioned ≥15 kb apart on the bacterial genome (Dingle *et al.*, 2001). Alleles termed *pgm* and *uncA* in the MLST scheme have been renamed *glmM* and *atpA* respectively in later genome annotations, but their names have been retained within the MLST scheme, for consistency with previous studies. The alleles from 2953 distinct STs were analysed.

### Identification of mosaic alleles.

Candidate mosaic alleles were identified using the model-based clustering algorithm implemented in the software structure (Pritchard *et al.*, 2000). All the alleles were analysed for each of the seven MLST loci to identify the genetic ancestry of the allelic variants. A population number (*k*) of 2 was used. This ensured that the analysis robustly identified putative ancestry to the two species (*C. jejuni* and *C. coli*), which are approximately 12 % divergent at the nucleotide level. Alternative values for *k*, for example *k*=4 to reflect the three-clade structure within *C. coli*, were not used because the nucleotide identity within *C. jejuni* is >98.5 % and among the three *C. coli* clades it is >93 %. Therefore, variation between MLST alleles within these two species is generally <10 polymorphisms, compared to >50 between species. This affects the robustness of population assignment using structure. Alleles where the probability of belonging to either the *C. jejuni* or the *C. coli* population was ≤0.75 were considered as possible mosaic alleles. A cut-off of 0.75 was chosen to conservatively detect potential mosaic alleles.

### Recombinant fragment characterization.

structure-based analysis was used to confirm whether possible hybrids identified were mosaic alleles. This involved the characterization of potential mosaic alleles using a linkage model (Falush *et al.*, 2003), which allowed for disequilibrium in linkage between loci, in this case nucleotides, and enabled the identification of the position of inter-specific recombination events. A comparison dataset (*n*=194), which described the diversity of *C. jejuni* (122) and *C. coli* (72) STs in the pubMLST database (Jolley *et al.*, 2004), was analysed alongside the STs containing mosaic alleles (*n*=81). The population of origin (POPINFO) was assigned for all the STs in the input file, but only the non-recombinant isolates were used, as a training dataset (POPFLAG), to define the background population structure. Origin-population was assigned probabilistically for each nucleotide (3309 bp) in all of the STs including those containing mosaic alleles. 10 000 burn-in cycles were run with 10 000 additional repetitions for all the analysis. Otherwise, the no admixture model was used with default settings.

### Clonal relationships.

The genealogy of the STs was estimated using clonalframe, a model-based approach to determining microevolution in bacteria (Didelot & Falush, 2007), which calculates clonal relationships with improved accuracy as it distinguishes point mutations from imported chromosomal recombination events, which are the source of the majority of allelic polymorphisms. clonalframe analysis was carried out on concatenated sequences of the same 275 STs as used in the structure analysis (above). The program was run with a burn-in of 50 000 burn-in iterations followed by 50 000 data collection iterations. The consensus tree represents combined data from three independent runs with 75 % consensus required for inference of relatedness.

### Origin of allelic recombination.

The origin of the largest putatively imported region within each mosaic allele was determined using the blast algorithm (Altschul *et al.*, 1990). Sequences were compared to a library database of all the non-recombinant alleles at that locus and the origin was assigned based on the highest identity (%) of the longest possible alignment region. Mosaic alleles, and the origin of recombination determined using blast, were indicated on phylogenies of individual alleles reconstructed using mega software, version 3.1, using the Kimura two-parameter model and neighbour-joining clustering (see Supplementary Fig. S1, available with the online version of this paper).

### Structuring of species and gene flow.

The number of fixed differences, shared polymorphisms and between-population gene flow – estimated by *F*_ST_ – were calculated using the DnaSP V 4.0 (Rozas *et al.*, 2003) and Arlequin V 3.1 (Excoffier *et al.*, 2005) software packages. Formal species assignment of STs was carried out as previously described (Sheppard *et al.*, 2008) using structure, with a threshold probability of 0.75 (75 %) being used as the cut-off for membership of a particular ST to each species, and clonalframe for clade assignment of *C. coli* haplotypes. Combining these data enabled the assignment of each ST, including those containing mosaic alleles, to a given species/clade for quantitative analysis of gene flow between groups.

### Micro-evolutionary analysis of polymorphisms.

Analysis was carried out to investigate the polymorphic sites within host and donor regions of mosaic alleles in the four largest mosaic allele clusters (see Fig. 3). Polymorphic sites were characterized within the recombinant and non-recombinant portions of each mosaic allele using start2 (Jolley *et al.*, 2001) and mega (Kumar *et al.*, 2004) software, and two analyses were carried out to investigate the polymorphisms within *C. coli* and *C. jejuni* alleles. First, individual polymorphisms within recombinant regions were compared to those in non-recombinant alleles and the synonymous and non-synonymous nucleic acid substitutions were quantified. Second, the Baysian analysis software structure was used to probabilistically assign polymorphisms within recombinant regions to *C. jejuni* or *C. coli* to determine if the conserved polymorphisms were putatively more similar to those associated with one of the two species. In some cases there were low numbers of nucleotide polymorphisms within the MLST alleles; for example there were only two nucleotide differences between the mosaic alleles *aspA*-87 and *aspA*-117, which limited the extent to which inference could be made.

## RESULTS

### Mosaic allele characterization

**Fig. 1.**
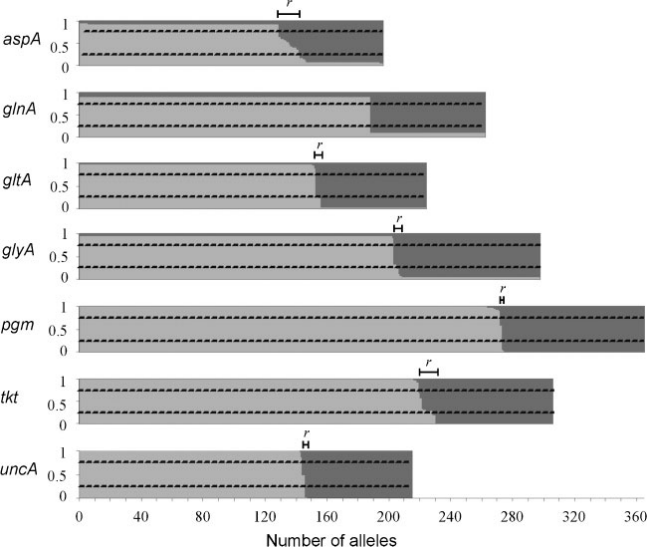
Identification of mosaic alleles: cluster analysis using structure inferring the probability-based genetic ancestry for allelic variants of the seven MLST loci (*aspA*–*uncA*). Each unique allele sequence is represented with vertical lines, divided into two shaded regions indicative of genetic ancestry to *C. jejuni* (light grey) or *C. coli* (dark grey). From this information inter-specific recombination between these species can be inferred. Alleles not assigned to a single genetic ancestry (*P*≥0.95), and with assignment probability ≤0.75, are considered inter-genomic recombinants (*r*). The analyses were carried out with *k*=2.

From the 2953 STs examined, which contained 1738 alleles, a total of 31 alleles were defined as mosaics on the basis of population assignment probabilities of ≤0.75 to groups corresponding to either *C. jejuni* or *C. coli* (Fig. 1). Further analysis identified putative recombination points in each mosaic which were consistent with mixed ancestry. The greatest number of mosaic alleles was recorded in the *aspA* locus (12 mosaic alleles) and *tkt* locus (11 mosaic alleles). There were three alleles defined as mosaic in the *gltA* and *glyA* loci and one in the *pgm* and *uncA* loci. No evidence was found for mosaic alleles at the *glnA* locus. The 31 mosaic alleles were distributed among 81 STs. Sixteen mosaic alleles were specific to one ST, 11 were found in two to five STs, mosaic alleles *aspA-*87 and *gltA*-134 were each found in 8 STs, *tkt-*12 was in 11 STs and *tkt*-169 was in 12 STs.

### The origin of recombinant regions

The distribution of mosaic genes was asymmetrical between *C. jejuni* and *C. coli*, with six of the mosaic alleles representing horizontal gene transfer from *C. coli* into *C. jejuni* STs and the remaining 25 from *C. jejuni* into *C. coli* STs. The direction of gene transfer, estimated by blast assignment of the recombinant regions, identified the ancestry of the recombinant region of the mosaic alleles (Supplementary Fig. S1). In alleles *aspA-*54, *aspA*-87, *aspA-*120, *glyA*-208, *glyA*-240 and *tkt-*166, this could be assigned to an identifiable *C. jejuni* lineage. The possible origin of the recombinant regions in the other mosaic alleles was more ambiguous because there was insufficient sequence variation in the derived allele to identify a particular donor lineage. However, recombination estimates were possible at the species or clade level and all but one episode of gene flow between *C. jejuni* and *C. coli* involved a single *C. coli* clade (clade 1). The exception was *tkt-*194, found in a single *C. jejuni* ST, which had regions of *C. jejuni* and *C. coli* clade 3 origin.

### Inter-species gene flow

Quantification of mosaic alleles allowed the investigation of patterns of gene flow. Nineteen (0.86 %) of the 2221 *C. jejuni* STs contained mosaic alleles and 105 (16.56 %) of the 634 *C. coli* STs contained mosaic alleles. No mosaic alleles were found among STs from *C. coli* clades 2 and 3. Patterns of allele distribution, quantified based on the proportion of the total number of alleles and the blast-defined origin of the recombinant fragment, gave a conservative estimate of gene flow (Table 1). *C. coli* had acquired DNA from *C. jejuni* in 8.3 % of alleles, 17 times more prevalent than the reciprocal process (0.5 % of *C. jejuni* alleles were mosaics).

### Clonal structure and allele mosaics

**Fig. 2.**
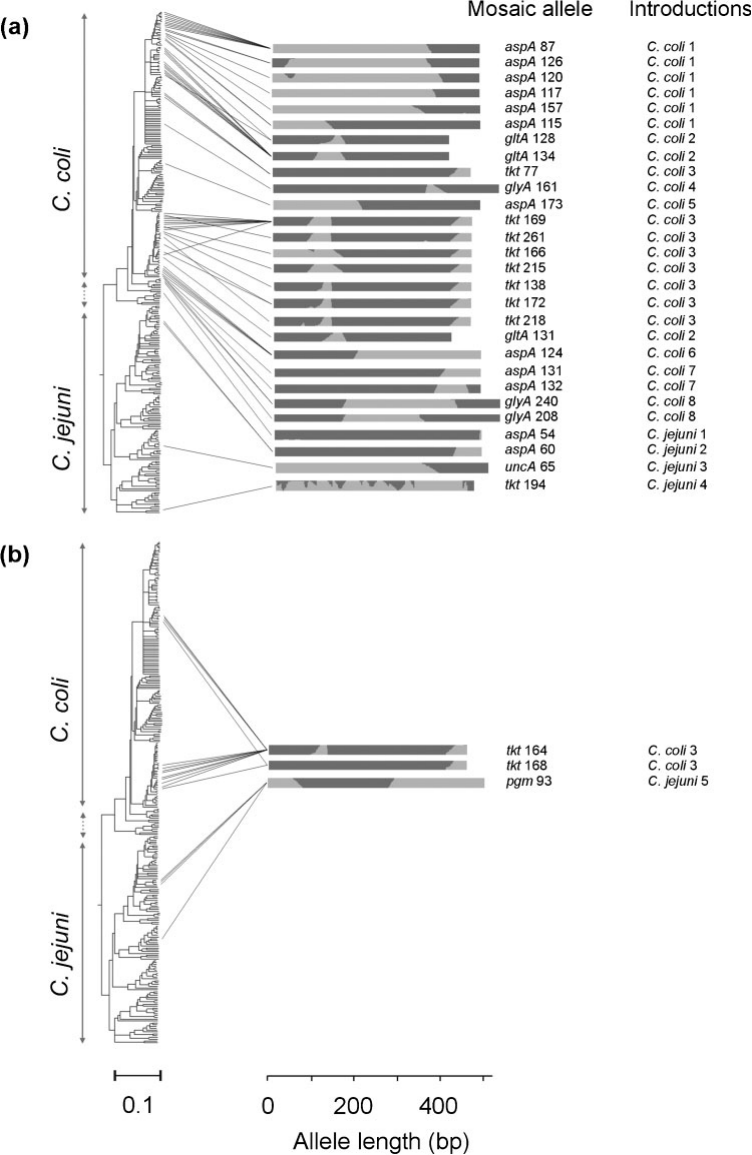
Distribution of mosaic alleles: consensus trees from clonalframe analysis of concatenated sequences of 275 STs from a combined *C. coli* and *C. jejuni* population, including 81 STs containing one or more inter-genomic mosaic alleles. Lines connect the mosaic alleles to the STs in which they occur. Alleles located in (a) closely related and (b) distant clades are indicated in separate trees. Site-by-site nucleotide ancestry, inferred using a linkage model in structure, is given for mosaic alleles describing putative origin within *C. jejuni* (light grey shading) and *C. coli* (dark grey shading). Background population structure (*k*=2) was defined using non-recombinant STs. The number of founding introductions was estimated from shared patterns of mosaicism among alleles and the clustering of mosaic alleles on the tree.

**Fig. 3.**
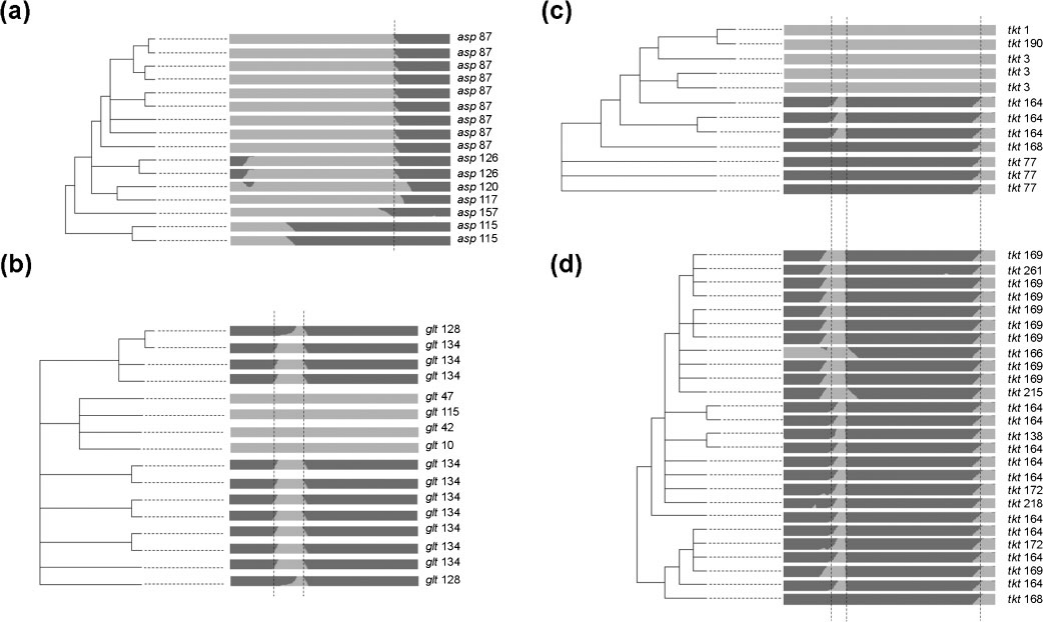
Major mosaic allele clusters: enlarged clonalframe tree topologies and mosaic allele intra-allelic recombinant regions from the four largest ST clusters containing mosaic alleles of the *aspA* (a), *gltA* (b) and *tkt* [*tkt*1 (c) and *tkt*2 (d)] loci. Site-by-site putative nucleotide ancestry is given for the numbered mosaic alleles (*C. jejuni*, light grey shading, *C. coli*, dark grey shading), and the approximate terminal positions of recombinant fragments are indicated with broken lines.

A clonalframe genealogy, generated with concatenated MLST gene sequences, partitioned *C. jejuni* and *C. coli* into two distinct groups, which was consistent with the *F*_ST_ values of 0.86 (DnaSP) and 0.87 (Arlequin) calculated from the same data. Eleven STs containing mosaic alleles were found within *C. jejuni* and 70 in *C. coli* clade 1. The mosaic alleles were examined in relation to this genealogy (Fig. 2). Mosaicism could be indicative of non-contiguous imports, as has been observed in *Helicobacter pylori* (Kulick *et al.*, 2008), but shared patterns of mosaicism among alleles and the clustering of mosaic alleles on the tree are likely to be indicative of single founding events followed by subsequent expansion. Based on this assessment there was evidence for 13 introductions, five among *C. jejuni* and eight among *C. coli* (Fig. 2). Recombination events between *C. jejuni* and *C. coli* had varying levels of subsequent clonal expansion, indicated by clusters of related STs containing the same mosaic allele. For example, the putative introduction into *C. coli*, termed ‘*coli* 1’, which generated alleles *aspA*-87, *aspA*-126, *aspA*-120, *aspA*-117, *aspA*-157 and *aspA*-115, had expanded into 16 clonally related lineages (Figs 2 and 3). There was also evidence of horizontal transfer of mosaic alleles following initial founding events within both *C. jejuni* and *C. coli*. The mosaic *pgm*-93 allele was found in two distantly related *C. jejuni* lineages, and *tkt*-164 and *tkt*-168 were both found in the two largest *C. coli tkt* mosaic allele clusters (Figs 2 and 3). In all ST clusters that contained more than one mosaic allele, the recombination pattern appeared non-random with shared terminal ends, indicative of subsequent recombination events after the acquisition of a mosaic allele. For example, in the two main clusters of *tkt* mosaic alleles, the uniformity of the terminal position of the recombinant regions was consistent with whole-allele replacements being subsequently eroded by reacquisition of *C. coli* DNA (Fig. 3).

### Micro-evolution of mosaic alleles

Some indication of possible genetic events in the evolution of the mosaic genes may be evident from patterns of sequence polymorphism within them. However, in this study the strength of these inferences was constrained by the limited number of polymorphisms within the mosaic alleles. The most parsimonious explanation for genetic relatedness within the four largest mosaic allele clusters (*aspA*, *gltA*, *tkt1*, *tkt2* – Fig. 3) was a single founding recombination event followed by subsequent clonal expansion accompanied by diversification due to mutation and recombination events. Within these mosaic allele clusters (Fig. 3), the total numbers of non-synonymous (*n*) and synonymous (*s*) base substitutions in all the recombinant regions in each cluster were 11, 171 (*aspA*); 2, 8 (*gltA*); 7, 12 (*tkt1*); and 26, 49 (*tkt2*). The number expected if the whole allele had been replaced would be 5, 46 (*aspA*); 8,33 (*gltA*); 14,45 (*tkt1*); and 14,45 (*tkt2*). The *n/s* values for recombinant regions were therefore higher than the average for a whole-allele replacement in the *gltA* (0.25>0.24), *tkt1* (0.58>0.31) and *tkt2* (0.53>0.31) clusters. However, although the observed cross-species imports in these examples had a higher proportion of synonymous changes than the part of the allele that was not present, the number of polymorphisms was low, and comparison of much larger sequence tracts would be necessary to confirm if this is because regions with a high proportion of non-synonymous mutations were not imported or, more probably, if they were eroded to a greater extent by later events. Similarly, while assignment of individual polymorphisms within the mosaic alleles with structure indicated that the recombinant regions had a greater mean assignment probability to *C. coli* than the polymorphisms in the eroded regions for the *aspA* (0.11>0.1), *gltA* (0.33>0.08), *tkt1* (0.24>0.09) and *tkt2* (0.18>0.0.07) mosaic allele clusters, the number of polymorphisms was too low to test if imported *C. jejuni* DNA that remained in *C. coli* alleles was more similar to the original *C. coli* sequence than that which had theoretically been removed.

## DISCUSSION

The patterns of mosaic alleles in *C. jejuni* and *C. coli* clade 1 were consistent with a recent increase in gene flow between these two organisms, as suggested by the analysis of whole-allele replacements of housekeeping genes reported previously (Sheppard *et al.*, 2008). As in the whole-allele analysis, which excluded mosaic alleles to ensure a conservative estimate of gene flow, the distribution of mosaic alleles is consistent with frequent introgression of housekeeping genes, or fragments of housekeeping genes, from *C. jejuni* into *C. coli* clade 1. The interpretation of the whole MLST allele analysis has been challenged in line with the view that horizontal genetic exchange of housekeeping genes must be infrequent in the absence of strong positive selection or hitchhiking (Caro-Quintero *et al.*, 2009). However, the analysis of Caro-Quintero *et al.* (2009) did not use a formal population-genetic approach for assigning species and clade designations, and did not identify the clades within *C. coli*, significantly altering the estimates of gene flow obtained. These alternative estimates were further affected by the use of the catalogue of ST and allele variation present in the PubMLST database as a representative population sample, which it is not. In addition, some hybrids that may have expanded clonally were excluded from the analysis, without a reciprocal exclusion of non-hybrids that may have done so, further altering the impact of introgression, by decreasing the relative proportion of hybrid lineages. Finally, ST data that did not conform to the assumption of low genetic exchange among housekeeping genes were dismissed as clerical errors. Although no evidence was presented to substantiate this assertion, this last criticism is met by the analysis of mosaic genes alleles, as such clerical errors are not possible and the most likely explanation of mosaic alleles is that they are the product of inter-species recombination. In conclusion, the data presented here on mosaic genes are supportive of the original analysis, which proposed a recent increase in gene flow between *C. jejuni* and *C. coli* clade 1 that, if sustained, will lead to progressive convergence or despeciation of *C. coli* clade 1, but not identifiably *C. coli* clades 2 and 3, with *C. jejuni* (Sheppard *et al.*, 2008).

The levels of introgression obtained from the analysis of mosaic genes were similar, but not identical, to those obtained from whole MLST allele analysis. Within *C. coli* clade 1, 105 STs (17 %) and 25 unique alleles (8 %) contained mosaic regions of *C. jejuni* ancestry (Table 1). The latter value is somewhat lower than the estimate of 18 % introgression based on whole-gene analysis. This is not inconsistent with the earlier findings; however, the MLST data for these organisms suggest that the average recombination fragment size is considerably larger than the average size of the of the sequences used to define MLST alleles (402–507 bp) (Sheppard *et al.*, 2008; Wilson *et al.*, 2009): therefore much of the variation is the result of reassortment of existing alleles (Dingle *et al.*, 2001; Wilson *et al.*, 2009) and analysis of mosaic alleles is likely to underestimate introgression from *C. jejuni* to *C. coli*. In addition, the method for defining mosaic alleles ignores alleles where <25 % of DNA is introgressed and therefore the estimate of 8 % of *C. coli* genes representing introgression events from *C. jejuni* is conservative for this dataset. It is noteworthy that the number of unique introgression events between *C. jejuni* and *C. coli* clade 1 appears to be similar in both directions (eight from *C. jejuni* to *C. coli* clade 1; five from *C. coli* to *C. jejuni*) so that the differences in the influence of the introgressed DNA in each species is apparently dependent on the fate of the hybrids and mosaics after these events have occurred. Indeed there is only one common example of the persistence of *C. coli* clade 1 DNA in *C. jejuni*, that of the *uncA*-17 allele in ST-61 clonal complex isolates. This contrasts with the large amounts of *C. jejuni* DNA within *C. coli* clade 1 STs (Sheppard *et al.*, 2008) and alleles reported here.

It is likely that mosaic alleles are continually generated among closely related transformable bacteria, as evidenced by many anecdotal reports of their occurrence (Dingle *et al.*, 2005; Feil *et al.*, 1995; Zhou & Spratt, 1992; Zhou *et al.*, 1997), but remain at low frequency. In some cases mosaic genes spread within a population as a result of positive selection for a novel adaptive phenotype that they confer. A well-established example of this is the spread of mosaic genes encoding penicillin-binding proteins, which are associated with increased penicillin resistance in a number of bacteria (Coffey *et al.*, 1995; Dowson *et al.*, 1990). For pathogenic and commensal bacteria selection pressures may be different at different stages in transmission. For example, in the species *Neisseria meningitidis*, bacteria with mosaic antigen-encoding genes generated within hosts by interspecies genetic exchange, apparently experiencing an advantage in that host as a consequence of the host immune response, appear to be less fit in onward human-to-human transmission such that the hybrids are subsequently lost (Zhou *et al.*, 1997). The extent to which stabilizing selection affects the spread of mosaic housekeeping genes remains unclear, but reduced selection against introgressed genes and mosaic genes could explain the observed increase in the proportion of whole-gene replacements and mosaic *C. jejuni* genes in *C. coli* clade 1. The patterns of polymorphisms within the mosaics are broadly supportive of a role of some selection against mosaics, but this is based on a small number of polymorphisms, and more data are required to investigate this possibility more rigorously.

The patterns of variation across bacterial genomes in features such as gene order, distribution of coding sequences on leading and lagging strands, GC skew, and codon usage are consistent with selection operating on sequence features other than maintenance of the protein sequences encoded (Bentley & Parkhill, 2004). A number of studies have indicated pervasive selection pressures across much of the genome in *Escherichia coli* and *Salmonella enterica* (Charlesworth & Eyre-Walker, 2006) and the genus *Campylobacter* (Lefébure & Stanhope, 2009). It is possible that intensive agriculture has generated a novel niche in which both *C. jejuni* and *C. coli* clade 1 thrive, promoting genetic exchange between these two bacteria, with a concomitant change in selective pressure that favours or tolerates mosaic alleles and whole-gene hybrids, but this intriguing possibility requires much more investigation. With whole-genome analyses of multiple isolates becoming increasingly possible, such studies are now feasible, and the interactions of *C. jejuni* and *C. coli* clade 1 populations at the genomic level provide a model system for distinguishing between the various contrasting theoretical frameworks currently being proposed to explain the relative roles of selection, genetic exchange and neutral mechanisms in the evolution and maintenance of genetic structure in bacterial populations.

## Figures and Tables

**Table 1.** Predicted origin of mosaic alleles given for genotypes assigned to *C. jejuni* and *C. coli* clades 1 to 3

**Recombination with:**	**Percentage of total number of alleles (*n*)**			
	***C. jejuni* (1242)**	***C. coli* clade 1 (302)**	***C. coli* clade 2 (74)**	***C. coli* clade 3 (126)**
*C. jejuni*	91.72	8.28	0	0
*C. coli* clade 1	0.40	99.6	0	0
*C. coli* clade 2	0	0	100	0
*C. coli* clade 3	0.08	0	0	99.92

## Abbreviations

- MLST, multi-locus sequence typing
- ST, sequence type
